# Modulation of Inflammatory Reactions by Low-Dose Ionizing Radiation: Cytokine Release of Murine Endothelial Cells Is Dependent on Culture Conditions

**DOI:** 10.1155/2018/2856518

**Published:** 2018-06-03

**Authors:** Sabine Schröder, Stephan Kriesen, Daniel Paape, Guido Hildebrandt, Katrin Manda

**Affiliations:** Department of Radiotherapy and Radiation Oncology, University Medical Center Rostock, Südring 75, 18059 Rostock, Germany

## Abstract

**Background:**

In many European countries, patients with a variety of chronical inflammatory diseases are treated with low-dose radiotherapy (LD-RT). In contrast to high-dose irradiation given to tumor patients, little is known about radiobiological mechanisms underlying this clinical successful LD-RT application. The objective of this study was to gain a better insight into the modulation of inflammatory reactions after LD-RT on the basis of endothelial cells (EC) as major participants and regulators of inflammation.

**Methods:**

Three murine EC lines were cultivated under 2D and 3D culture conditions and irradiated with doses from 0.01 Gy to 2 Gy. To simulate an inflammatory situation, cells were activated with TNF-*α*. After LD-RT, a screening of numerous inflammatory markers was determined by multiplex assay, followed by detailed analyses of four cytokines (KC, MCP-1, RANTES, and G-CSF). Additionally, the monocyte binding to EC was analyzed.

**Results:**

Cytokine concentrations were dependent on culture condition, IR dose, time point after IR, and EC origin. IR caused nonlinear dose-dependent effects on secretion of the proinflammatory cytokines KC, MCP-1, and RANTES. The monocyte adhesion was significantly enhanced after IR as well as activation.

**Conclusions:**

The study shows that LD-RT, also using very low radiation doses, has a clear immunomodulatory effect on EC as major participants and regulators of inflammation.

## 1. Introduction

For the treatment of a variety of chronical inflammatory and painful joint diseases such as heel spurs [1] or osteoarthritis [2] as well as periarthritis humeroscapularis [3], low-dose radiotherapy (LD-RT) is practiced in many European countries [4, 5]. Total doses of LD-RT include 5% to 10% of those given to tumor patients, assuming different radiobiological mechanisms caused by LD-RT compared to high-dose radiotherapy (HD-RT). Whereas HD-RT used in cancer therapy has been proven to induce proinflammatory processes on the immune system [6], after LD-RT, anti-inflammatory and analgesic effects are clinically observed [2, 7, 8]. But the knowledge about radiobiological mechanisms underlying this clinical successful LD-RT application is limited [9]. Therefore, the objective of this study was to gain a better insight into the modulation of inflammatory reactions after LD-RT.

Major participants and regulators of inflammatory processes are known to be endothelial cells (EC) [10]. EC are involved in recruiting of immune cells from the peripheral blood (adhesion) to the inflammation side. Once activated by TNF-*α* or proinflammatory lipopolysaccharide, EC are responsible for the secretion of many chemokines, cytokines, and growth factors as well as adhesion molecules [11–14]. It was shown that their function is modulated also by irradiation [15]. Previous investigations already showed a reduced adhesion of peripheral blood mononuclear cells (PBMCs) to EC after LD-RT [16, 17]. Several other studies revealed the anti-inflammatory effect of LD-RT on various cells, for example EC, with different responses to the applied radiation doses [18]. In TNF-*α*-activated EC, a nonlinear expression and activity of compounds of the antioxidative system were described, assuming to contribute to the anti-inflammatory effect [8]. So on the basis of EA.hy926 EC and human umbilical vein endothelial cells (HMVECs), the authors showed a discontinuous expression and enzymatic activity of glutathione peroxidase accompanied by a lowered expression and DNA-binding activity of the transcription factor nuclear factor E2-related factor 2 (Nrf2) after LD-RT. But most of the *in vitro* studies investigating the effect of LD-RT on EC were performed using two-dimensional (2D) cultivation conditions.

In 2D cultivation, EC grow as a homogenous monolayer on different plastic or glass substrates which do not reflect the physiology of the *in vivo* situation. The morphology of cells as well as cell-cell and cell-matrix interactions are different in tissue or organs compared to flat 2D cell culture conditions [19, 20]. The use of eligible three-dimensional (3D) cell culture models facilitates a tissue or organotypic differentiation of cells. Mechanical and biochemical signals and reactions, communications between cells, or the surrounding matrix are better reestablished under 3D conditions. A deeper insight into migration or adhesion behavior of cells is given likewise in 3D models studying cell physiological reactions to stress-inducing stimuli or effects caused by IR and chemotherapeutic treatment of cells [21]. Experiments have shown differences in the expression patterns of various genes in melanoma cells [22] or human lung fibroblasts [23] as well as mammary epithelial cells [24, 25] when cultured in 3D compared to 2D. Former studies with prostate cancer cells did not only discover different response behaviors of cells; they also demonstrated differences in metabolism and differentiation when cells were cultured in the Matrigel™-based extracellular matrix (ECM) [24–26]. Experiments under 3D culture conditions thus shall give a more detailed picture of inflammatory reactions after exposure to IR, which is more closely reflective to the events *in vivo*. Therefore, for gaining a better insight into the modulation of inflammatory reactions after LD-RT, one main objective in the present study was to investigate the effect of LD-RT on the alteration in cytokine release of EC exposed to single doses of X-rays, with special emphasis on the differences between 2D and 3D culture conditions.

## 2. Materials and Methods

### 2.1. Cell Lines and Cultivation

All experiments were performed with three murine EC lines and one murine monocyte cell line. The mlEND1 cells present mesenterial lymph node-derived EC. The H5V cells, derived from the embryonic heart (described in [27]), were kindly provided by Dr. Annunciata Vecchi, Centro Ricerche, Istituto Clinico Humanitas, Rozzano, Italy. The cerebral cortex EC bEND3 was purchased from the American Type Culture Collection (ATCC, Manassas, VA, USA). The monocyte cell line WEHI-274.1 was purchased from the European Collection of Cell Cultures (ECACC, Salisbury, UK).

Cells were cultured in Dulbecco's modified Eagle's medium (DMEM, Lonza, Cologne, Germany) supplemented with 10% heat-inactivated fetal calf serum (FCS, Merck Millipore, Darmstadt, Germany), 100 U/mL penicillin, and 100 *μ*L/mL streptomycin (Sigma-Aldrich, Hamburg, Germany) in 75 cm^2^ flasks at 37°C and 5% CO_2_.

#### 2.1.1. 2D Cell Cultivation

For the two-dimensional cultivation, cells were seeded in 24-well plates (analysis of pro- and anti-inflammatory cytokines) or 96-well plates (viability assay) and cultured under standard conditions.

#### 2.1.2. 3D Cell Cultivation

For the three-dimensional cultivation, the multiwell plates were precoated with agarose, and cells embedded in growth factor-reduced Matrigel (BD Biosciences, Heidelberg, Germany—now sold by Corning Incorporated, NY, USA) with a 0.5 mg/mL protein concentration were plated on the agarose layer and cultured under standard conditions.

### 2.2. 2D Monocyte-Binding Assay

EC were seeded in duplicates in 6-well plates for 24 hours and cultured under standard conditions. Subsequently, the medium was replaced with serum-free medium supplemented with or without TNF-*α* 2 hours before irradiation. After irradiation, cells were kept in the incubator for 24 hours. The nonirradiated WEH1 monocytes were dyed with CFSE (Molecular Probes—now sold by Thermo Fisher Scientific, Schwerte, Germany), for 5 min in the incubator and then were added to EC for 2 hours (static adhesion assay) at 37°C/5% CO_2_, followed by several gentle washing steps to remove unbound monocytes. The number of monocytes bound to EC was determined by using the flow cytometer FC500 (Beckman Coulter, Krefeld, Germany).

### 2.3. TNF-*α* Stimulation

Prior to radiation, EC were activated with TNF-*α* (R&D Systems, Wiesbaden, Germany) to stimulate the secretion of inflammatory markers by simulating an inflammation. For that, 24 hours after seeding and 2 hours before IR, the medium was replaced by serum-free medium with or without supplementation of ng/mL TNF-*α*.

### 2.4. Ionizing Radiation (IR)

At 24 h after seeding, EC were irradiated with X-rays using an Xstrahl 200 therapy system (Xstrahl Ltd., Surrey, United Kingdom; dose rate 0.52 Gy/min and energy 200 kV) at room temperature with single doses of 0.01 Gy, 0.05 Gy, 0.075 Gy, and 0.1 Gy. Cells irradiated with 2 Gy served as positive control. Sham-irradiated samples (0 Gy) were kept at room temperature in the X-ray control room while others were irradiated.

### 2.5. Analysis of Cellular Metabolic Activity

The metabolic activity of the cells was detected by the water-soluble tetrazolium 1 (WST-1) assay (Roche Deutschland GmbH, Mannheim, Germany) 4 hours, 24 hours, and 48 hours after IR. Cell samples were seeded in triplicates in 96-well plates, TNF-*α* activated, and grown under standard conditions (see above). After IR, WST-1 reagent was added for 2 hours and the assay's specific instruction was followed for analysis. The tetrazolium salt WST-1 was metabolized into a formazan dye solely by metabolically active cells. The number of vital cells directly correlated with the metabolized formazan dye quantity. The Anthos Zenyth 340r reader (Anthos Mikrosysteme GmbH, Krefeld, Germany) was used for spectrophotometric measurement.

### 2.6. Analysis of Cytokines

#### 2.6.1. Sample Collection for Cytokine Measurement

For collection of the supernatant, cells were seeded in 24-well plates and cultivated to confluence for 24 hours under standard conditions. 2 hours before IR, the medium was replaced by serum-free medium with or without addition of TNF-*α*. Cells were treated with radiation using doses ranging from 0.01 Gy to 2 Gy. 30 min and 48 hours after radiation, supernatants were collected and stored at −80°C until measurement of inflammatory cytokines. Samples without cells and only with agarose and Matrigel layer served as a control to assess the system.

#### 2.6.2. Measurement of Inflammatory Cytokines

Cytokine levels of 23 different inflammatory markers (IL-1*α*, IL-1*β*, IL-2, IL-3, IL-4, IL-5, IL-6, IL-9, IL-10, IL-12p40, IL-12p70, IL-13, IL-17A, eotaxin, G-CSF, GM-CSF, IFN-*γ*, KC, MCP-1, MIP-1*α*, MIP-1*β*, RANTES, and TNF-*α*) were quantified (data not shown) in supernatants harvested from EC cultivated in 2D as well as 3D models stimulated either with TNF-*α* or not by using a 23-plex mouse cytokine/chemokine kit from Bio-Rad Laboratories GmbH (Munich, Germany) according to the manufacturer's protocol. After measurement and evaluation, four cytokines with the most significant changes in cytokine expression levels were reexamined in more detail: G-CSF, KC, MCP-1, and RANTES with a customized 4-plex mouse cytokine/chemokine kit (Bio-Rad Laboratories GmbH). Data were acquired using the Bio-Plex® 200 suspension array system and analyzed with the Bio-Plex Manager™ Software (version 4.1).

### 2.7. Analyses of Relative MCP-1 Expression

#### 2.7.1. RNA Isolation

Total RNA was isolated 4 hours, 24 hours, and 48 hours post-IR from mlEND.1 by using the NucleoSpin® RNA kit (Macherey-Nagel, Duren, Germany) according to the manufacturer's protocol. The purity of the isolated RNA was verified using the Eppendorf BioPhotometer plus (Eppendorf AG, Hamburg, Germany) with 260/280 and 260/230 ratios as well as real-time PCR with RNA as template to verify the lack of genomic DNA contaminations causing false-positive results during amplification.

#### 2.7.2. Reverse Transcription

Less than 1 *μ*g of total RNA was reverse transcribed into cDNA using the RevertAid First Strand cDNA Synthesis Kit (Fermentas/Thermo Fisher Scientific, Schwerte, Germany) following the manufacturer's instructions.

#### 2.7.3. Quantitative Real-Time PCR

The cDNA was subsequently used in a 20 *μ*l real-time PCR containing TaqMan® Universal PCR Master Mix and TaqMan Gene Expression Assays for monocyte chemoattractant protein-1 (MCP-1) as well as glyceraldehyde 3-phosphate dehydrogenase (GAPDH; Life Technologies, Darmstadt, Germany) as an internal control and 36 ng of cDNA as template. Quantitative real-time PCR was carried out using the 7300 Real-Time PCR System (Applied Biosystems®, Life Technologies, Darmstadt, Germany). All reactions were performed in triplicates. The MCP-1 mRNA expression was normalized to the housekeeping gene GAPDH, which showed no change in expression profile after IR in pretests and therefore served as an internal control. To estimate the relative expression change, all samples were normalized to the control samples at 0 Gy for each time point which were set to 1 using the delta-deltaC_T_ (2^−CT^) method (comparative C_T_ method).

### 2.8. Statistical Analysis

All data are presented as means ± standard deviation (SD) on the basis of at least three independent experiments. The statistical significance of differences was assessed by the Student *t*-test. A value of *p* < 0.05 was considered as statistically significant.

## 3. Results

### 3.1. Influence of LD-RT on Metabolic Activity

The metabolic activity was measured in the three murine EC lines mlEND.1, H5V, and bEND.3 at 4 hours, 24 hours, and 48 hours after IR with different doses. The metabolic activity increased in all tested EC lines over time, but remained unaffected by radiation treatment or activation of the cells with TNF-*α* prior to IR (Supplement Figure 1). Therewith, all subsequent observed changes in secretion of different markers will be resulted by IR, not by alteration of metabolic activity.

### 3.2. Effect of LD-RT on Cytokine Secretion

An initial screening of cytokines should ascertain which of the 23 inflammatory markers (IL-1*α*, IL-1*β*, IL-2, IL-3, IL-4, IL-5, IL-6, IL-9, IL-10, IL-12p40, IL-12p70, IL-13, IL-17A, eotaxin, G-CSF, GM-CSF, IFN-*γ*, KC, MCP-1, MIP-1*α*, MIP-1*β*, RANTES, and TNF-*α*) were secreted by EC with and without IR and TNF-*α*. Levels of 23 inflammatory cytokines were quantified in supernatants of three different EC lines. From these 23 measured markers, 18 were below the detection limit of the assay or not detectable at all. Four out of 23 cytokines showed significant changes after IR: keratinocyte-derived chemokine (KC), monocyte chemoattractant protein-1 (MCP-1 or CCL2), regulated on activation, normal T cell expressed and secreted (RANTES or CCL5), and granulocyte-colony stimulating factor (G-CSF).

#### 3.2.1. Effect of LD-RT on Secretion of KC

**Figure 1:**
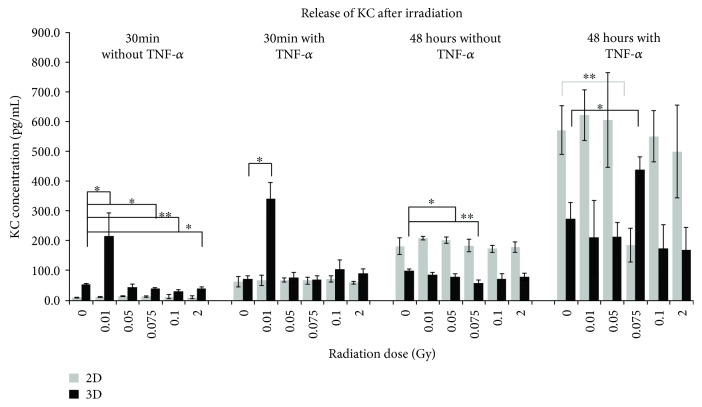
Released levels of the keratinocyte-derived chemokine (KC) in supernatant of H5V endothelial cells. The cytokine concentration was determined by multiplex assay at two time points after irradiation with low doses of X-rays. Changes in cytokine concentrations are presented as mean (pg/mL) ± standard deviation (SD) from three independent experiments; asterisks illustrate significance: ^∗^ *p* < 0.05 and ^∗∗^ *p* < 0.01.

Secretion of KC from the murine H5V cells was detected from 9.9 pg/mL to 623.4 pg/mL in 2D and 29.4 pg/mL to 439.0 pg/mL in 3D (Figure 1). The most significant changes were observed 30 min after IR in 3D-cultured cells without TNF-*α* supplementation at doses of 0.01, 0.075, 0.1, and 2 Gy and with TNF-*α* at a dose of 0.01 Gy. It is clearly to be seen that the irradiation dose of 0.01 Gy caused a significant increase in accumulation of KC in the supernatant of the cultured cells. Activation of the cells prior to IR enhanced the effect. The concentration of the cytokine was higher in 3D-cultured cells compared to the 2D samples. In contrast to these results, the KC values were higher in 2D-cultured cells 48 hours after IR. Without TNF-*α* only in the 3D samples, a significant reduction of KC was detectable at doses of 0.05 and 0.075 Gy. The addition of TNF-*α* before IR treatment caused an increase in both 2D and 3D samples with a significant reduced concentration in the 2D samples at 0.075 Gy, but a significant increase in the 3D samples at the same irradiation dose.

In mlEND.1 cells, the KC concentration in each experimental approach was similar, independently from time point, culture condition, or TNF-*α* (3D) and time-dependently increasing under 2D conditions (Supplement Figure 2A). In the bEND.3 cells, an increase from 30 min after IR without TNF-*α* to 48 hours after IR was observed under both culture conditions without significant changes (Supplement Figure 2B).

#### 3.2.2. Effect of LD-RT on Secretion of MCP-1

**Figure 2:**
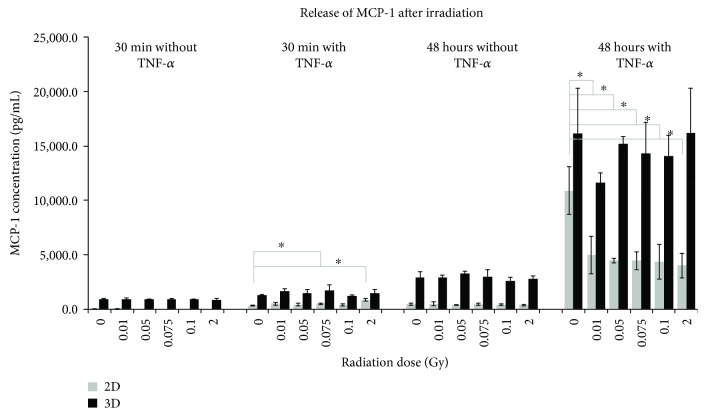
Released levels of monocyte chemoattractant protein-1 (MCP-1) in supernatant of bEND.3 endothelial cells. The cytokine concentration was determined by multiplex assay at two time points after irradiation with low doses of X-rays. Changes in cytokine concentrations are presented as mean (pg/mL) ± standard deviation (SD) from three independent experiments; asterisks illustrate significance: ^∗^ *p* < 0.05.

Secretion of MCP-1 from the murine bEND.3 cells was detected from 37.5 pg/mL to 10,898.2 pg/mL in 2D and from 854.6 pg/mL to 16,212.3 pg/mL in 3D (Figure 2). It is clearly to be seen that the accumulation of MCP-1 was highest under all experimental approaches when cells were cultured in 3D compared to the samples from 2D. The addition of TNF-*α* before IR caused no significant increase in concentration 30 min after irradiation, but 48 hours after, the MCP-1 concentration reached a maximum in the 3D samples, but without significant change. Compared to these results, cells from the 2D condition secreted MCP-1 significantly higher 30 min after IR at doses of 0.075 Gy and 2 Gy compared to 0 Gy, when TNF-*α* was supplemented. In contrast to this result, the MCP-1 secretion was significantly reduced 48 hours after IR at all tested doses compared to the sham-irradiated samples under the same culture conditions.

In H5V cells, a time- and TNF-*α*-dependent increase in MCP-1 was observed, but with no significant changes in 2D. Under 3D conditions, the effect was less distinctive, but with very significant changes 30 min after IR at a dose of 0.01 Gy. TNF-*α* supplementation enhanced this effect (Supplement Figure 3A). The mlEND.1 cells secreted MCP-1 in a time-dependent manner under 2D as well as 3D conditions, but only 30 min after IR, a significant change was visible (Supplement Figure 3B).

#### 3.2.3. Effect of LD-RT on Secretion of RANTES

**Figure 3:**
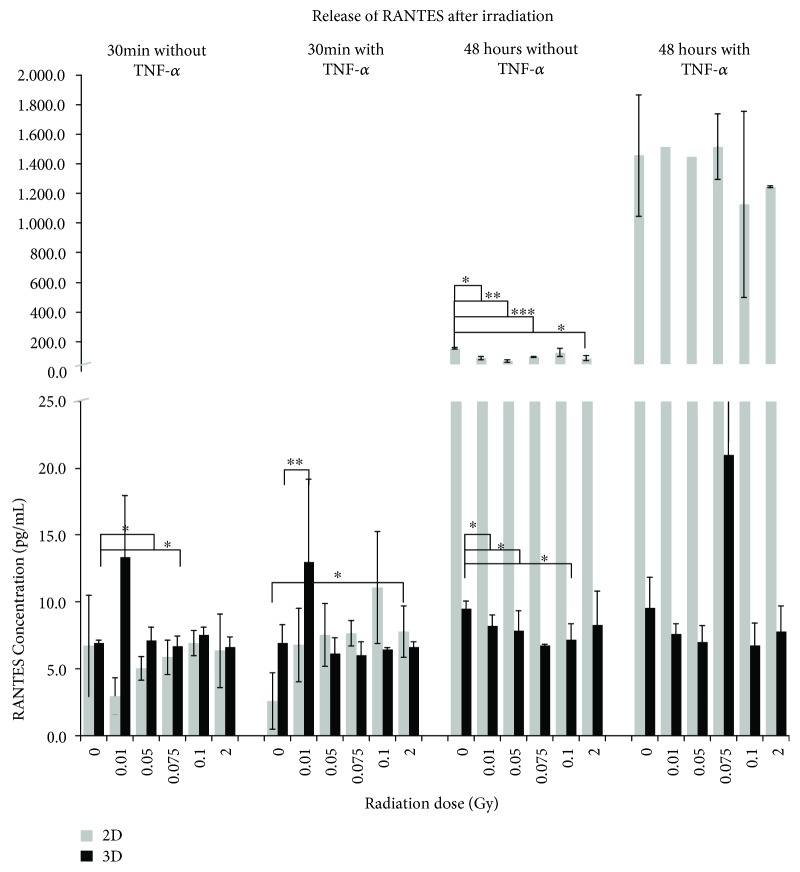
Released levels of RANTES in supernatant of mlEND.1 endothelial cells. The cytokine concentration was determined by multiplex assay at two time points after irradiation with low doses of X-rays. Changes in cytokine concentrations are presented as mean (pg/mL) ± standard deviation (SD) from three independent experiments; asterisks illustrate significance: ^∗^ *p* < 0.05, ^∗∗^ *p* < 0.01, and ^∗∗∗^ *p* < 0.001.

Secretion of RANTES1 from the murine mlEND.1 cells was detected in a range from 2.6 pg/mL to 1515.2 pg/mL in 2D and from 18.3 pg/mL to 53.6 pg/mL in 3D (Figure 3). RANTES was secreted by these cells under 2D conditions in a time-dependent manner with a maximum of concentration 48 hours after IR and additional TNF-*α*. Significant reduction was observed at this time point, but without TNF-*α* compared to the 0 Gy sample. When cells were cultured in a 3D system, the RANTES accumulation in the supernatant was not as high as in the 2D samples; a clear time-dependent increase was also not observable. TNF-*α* supplementation only caused a significant higher secretion 30 min after IR at a dose of 0.01 Gy whereas without, significantly reduced concentrations were measured 30 min after IR with doses of 0.075 Gy and 48 hours after IR at doses of 0.01, 0.05, and 0.1 Gy.

In H5V cells, RANTES was secreted time-dependently; TNF-*α* did not enhance the effect significantly (2D). When cells were cultured in a 3D environment, the RANTES concentrations showed the same level as in 2D, without a significant time- or dose-dependent tendency (Supplement Figure 4A). In bEND.3 cells, the same effect was observed under 2D conditions, with a slightly higher concentration compared to H5V cells. Under a 3D culture environment, RANTES was secreted at a similar level without significant time- or dose-dependent changes (Supplement Figure 4B).

#### 3.2.4. Effect of LD-RT on Secretion of G-CSF

If cultivated with a conventional 2D system, only mlEND.1 cells 48 hours after IR and TNF-*α* treatment alone secreted G-CSF (Supplement Figure 5A). In the 3D model, however, G-CSF concentrations were measurable in the supernatants of mlEND.1 and bEND.3 cells (Supplement Figure 5A) at both time points after IR (mlEND.1: 6.67 pg/mL to 39 pg/mL; bEND.3: 4.41 pg/mL to 9.43 pg/mL), but the difference between nonactivated and activated samples was less distinctive 30 min after IR compared to 48 hours later and only with mlEND.1 cells was the level of G-CSF increased 48 hours after IR. Both cell lines secreted the inflammatory marker in a nonlinear dose-dependent manner. Significant changes were measured 48 hours after IR. In the supernatant of H5V cells, G-CSF was not detectable.

### 3.3. Effect of LD-RT on Monocyte Binding

**Figure 4:**
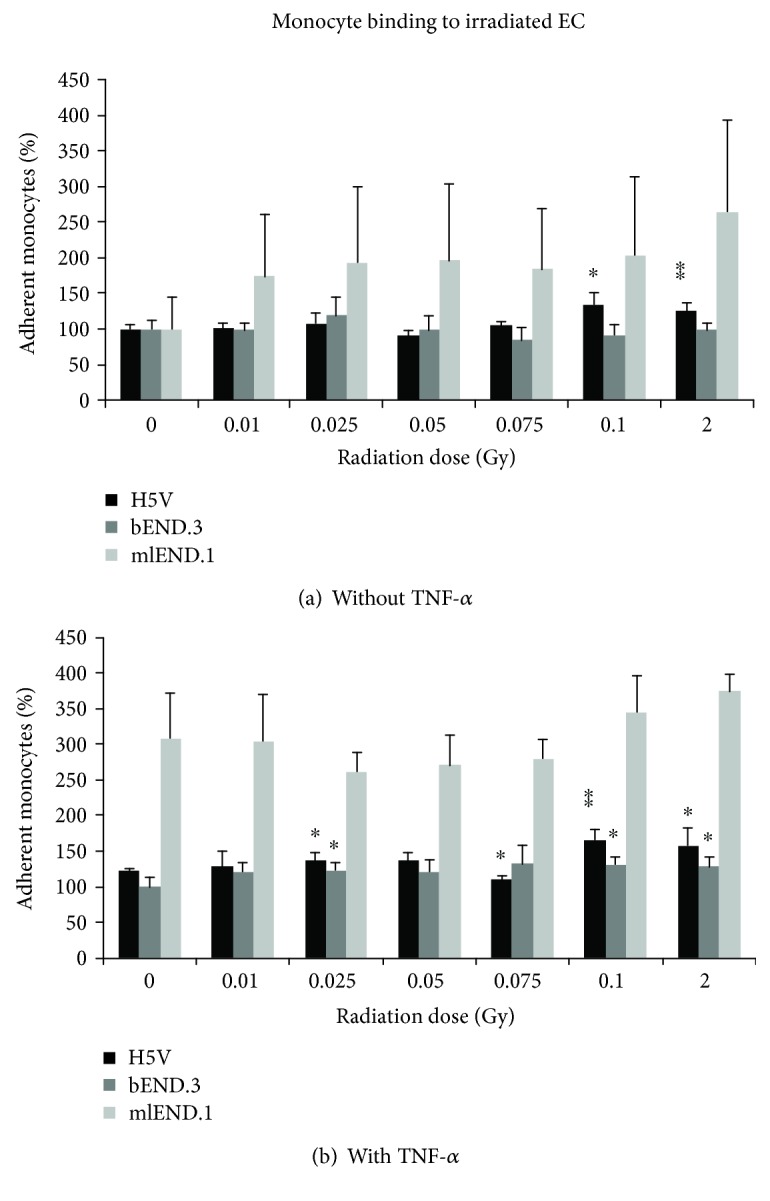
Adhesion of WEH.1 monocytes to irradiated EC (a) nonactivated and (b) activated with TNF-*α* prior to IR. The percentage adhesion of monocytes to EC was determined by flow cytometric analysis 24 hours after irradiation with low doses of X-rays and normalized to the sham-irradiated, nonactivated samples. Changes in monocyte binding are presented as mean ± standard deviation (SD) from four independent experiments performed in duplicates; asterisks illustrate significance: ^∗^ *p* < 0.05 and ^∗∗^ *p* < 0.01.

Sham-irradiated, nonactivated samples served as a control and were set to 100%. Monocytes remained nonactivated and nonirradiated. Under 2D cultivation conditions, the adhesion of the monocytes to nonactivated mlEND.1 cells resulted in a maximum level at 2 Gy, but with no significant influence of IR (Figure 4(a)). In nonactivated H5V cells, a significant higher adhesion after IR was detected at 0.1 Gy and 2 Gy compared to 0 Gy control. No significant differences in WEH.1 adhesion after IR were observed for nonactivated bEND.3 cells. The supplementation with TNF-*α* prior to IR resulted in an enhanced adhesion in all three cell lines (Figure 4(b)). With mlEND.1 cells, the adhesion was in average 200% higher compared to the nonactivated, sham-irradiated sample, but a significant influence of LD-RT was not detected. The adhesion of WEH.1 cells to H5V cells after IR was significantly higher at 0.025 Gy, 0.1 Gy, and 2 Gy as well as at 0.075 Gy compared to sham-irradiated, but activated samples. With bEND.3 cells, the adhesion after IR was significantly enhanced after doses of 0.025 Gy, 0.1 Gy, and 2 Gy. The 3D model used in this study for all other experiments—measurement of metabolic activity, cytokine secretion, as well as expression of MCP-1—was not applicable for investigation of monocyte binding to EC under 3D conditions after LD-RT.

### 3.4. Effect of LD-RT on Relative MCP-1 Expression

**Figure 5:**
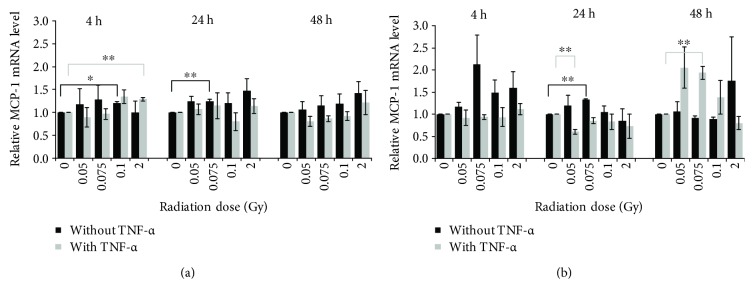
Relative MCP-1 mRNA expression level of (a) 2D- and (b) 3D-cultured mlEND.1 cells. The cytokine expression was determined by quantitative real-time PCR at three time points after irradiation with low doses of X-rays. Changes in cytokine expression are presented as mean (pg/mL) ± standard deviation (SD) from four independent experiments. The expression of MCP-1 was normalized to the housekeeping gene GAPDH; asterisks illustrate significance: ^∗^ *p* < 0.05 and ^∗∗^ *p* < 0.01.

To examine if the exposure of EC to LD-RT can not only alter the protein secretion but also influence the expression of inflammatory markers on an RNA level, a qRT-PCR for the MCP-1 gene was performed. Under 2D culture conditions, as shown in Figure 5(a), a significant upregulation of MCP-1 mRNA could be detected 4 hours and 24 hours after IR. A statistically significant expression change was not observed 48 hours postirradiation. The expression pattern of MCP-1 mRNA under 3D culture conditions differed from the pattern in the 2D model (Figure 5(b)). Without activation of the cells, a significant upregulation was detected 24 hours after IR at a dose of 0.075 Gy. In activated cells, the MCP-1 mRNA expression was significantly downregulated 24 hours after IR at a dose of 0.05 Gy and significantly upregulated again 48 hours after IR at a dose of 0.075 Gy.

## 4. Discussion

Cytokines and chemokines are known to contribute to all aspects of inflammatory reactions in different ways and interact with EC and other cells of the immune system [28–33]. The crosstalk between the molecules is of complex nature, and they can have an additive, synergistic, or antagonistic effect on the same process [34]. Among the 23 analyzed inflammatory markers in our study, four (KC, MCP-1, RANTES, and G-CSF) were found to be clearly altered. The results could demonstrate that the secretion of the different cytokines is mainly dependent on (I) the culture condition of the cells, (II) the origin of the cells, (III) the radiation dose, and (IV) the activation with TNF-*α*.

An alteration of metabolic activity as a reason of the observed changes in secretion of different markers could be excluded because of results from analyses of metabolic activity. We could demonstrate that the cell irradiation up to single doses of 2 Gy and until 48 hours after IR did not affect the metabolic activity significantly. Also, no significant changes were observed after activation with TNF-*α*. The results verified the preserved metabolic activity of all three EC after IR. The increase in metabolic activity was solely explained by normal cell growth over the observed time frame and therefore the gain of viable cells metabolizing the chemical. The cell division rate of the mlEND.1 cells is higher than the ones from bEND.3 and H5V. This method was used by many other authors like Cervelli et al. whose team irradiated human umbilical vein endothelial cells (HUVEC) with low-energy X-rays in single as well as fractionated low doses and also observed no significant loss of cell viability caused by IR [35].

One main goal of this study was the comparison of cytokine secretion from EC cultivated under 2D or 3D conditions and to investigate which culture system is more suitable to study the effect of LD-RT on EC. It was observed that altered releases of inflammatory cytokines were detectable in both culture models, 2D and 3D, but with clear differences. The proinflammatory marker G-CSF was clearly evident in the 3D model in mlEND.1 and bEND.3 cells. But under 2D conditions, G-CSF was measured only in mlEND.1 cells at very low concentrations. G-CSF plays a major role as regulator of haematopoiesis and innate immune responses and is well known to also promote angiogenesis and improves cardiac function [36, 37].

A key chemokine, which plays a pivotal role in the innate immunity, the pathogenesis of various infectious, and inflammatory diseases by attracting monocytes to the site of inflammation or injury, is the monocyte chemoattractant protein-1 (MCP-1) [38]. MCP-1 can be secreted by EC in response to signals like proinflammatory stimuli or IR. Therefore, this molecule is of high interest for low-dose IR research and how it contributes to the modulation of inflammatory reactions especially after LD-RT. In our study, the secretion of MCP-1 was also higher in 3D-cultured cells compared to the samples collected under 2D conditions. Using 2D conditions, the maximum concentration was measured in mlEND.1 and H5V cells in average, but highest in bEND.3 cells when cultured in a 3D model. The murine chemokine KC, a potent chemoattractant for neutrophils and involved in murine inflammatory processes [39], was secreted by mlEND.1 cells highest under 3D conditions, whereas in H5V and bEND.3 cells, 2D-cultured cells secreted this marker in highest concentrations. RANTES is a chemokine with inflammatory properties in a various number of tissues [40] and acts as a mediator of acute and chronic inflammation [29, 38]. All 3 cell lines secreted RANTES in the highest level in a 2D environment. In a study without IR, published by Ghosh et al., the expression of several genes in NA-8 cells (metastatic melanoma cells) was examined under 2D and 3D conditions, and a clear difference between both culture systems was also observed [22]. As an example, the expression of CXCL1 and IL-8 was significantly upregulated in the 3D model when compared to the 2D culture samples, as well as the secretion of the corresponding proteins. The expression of basic FGF, however, was downregulated. Cells in the organism are surrounded by tissue containing a variety of other cells and ECM. This allows the cells to communicate with neighboring cells as well as interact with the components of the ECM. In a conventional 2D experimental set-up, the tissue-specific architecture and biochemical and mechanical cues are lost and do not represent the correct *in vivo* conditions [41, 42]. To reestablish physiological interactions and a similar *in vivo*-like environment for the cells, many different 3D culture models were developed in recent years, including for investigation of radiation effects [19, 21]. Results received in this study were adapted from a Matrigel-based ECM cell culture model and compared to a conventional flat 2D cell culture model. Clear differences in the secretion of cytokines could be observed, not only depending on the culture environment but also depending on the origin of the cells.

For the present study, EC from different origins were used: embryonic heart (H5V—microvascular), mesenterial lymph node (mlEND.1—macrovascular), and frontal cortex (bEND.3—microvascular). The results showed that the secretory potential of EC after IR is dependent on the origin of the cells. In general, it could be observed that cells derived from large vessel (mlEND.1) responded with increased secretion of proinflammatory markers. Cells derived from the microvasculature (H5V) were affected less distinctive by LD-RT, secreted proinflammatory markers in lower concentrations compared to other EC, or did not secrete them at all.

It is well known that the adhesion of leukocytes to the endothelium is one of the initial events during tissue invasion and therefore contributes to inflammatory reactions. This event is mediated by various molecules [43, 44]. Several studies already revealed the reduced adhesion of peripheral blood mononuclear cells (PBMCs) to EC after LD-RT [16, 17]. In the study by Rödel et al., the adhesion of PBMCs to activated EA.hy926 EC cells was significantly reduced 24 hours after IR with 0.5 Gy [16].

Kern et al. published a study in 2000, where they examined if the adhesion capacity of murine EC (mlEND.1) is influenced by their activation status during IR [17]. The adhesion of PBMCs decreased by about 20–30% 24 hours after IR with 0.1 Gy and 0.5 Gy. In our study, we could show that the adhesion of monocytes to nonactivated EC not only was influenced by LD-RT alone but also was enhanced when cells were activated before IR. In contrast to the experimental set-up used in the studies mentioned above, we used X-rays in a very low-dose range of 0.01 Gy to 0.1 Gy as well as 2 Gy as the highest dose.

The 3D model used in this study for measurement of metabolic activity, analyses of cytokine secretion, as well as expression of MCP-1 was not transferable to investigate the monocyte binding to EC after LD-RT. The system used in this study was based on Matrigel with a concentration of 0.5 mg/mL protein. In the experimental set-up, it was not distinguishable if the monocytes were completely migrated through the ECM layer to bind to the EC or if the cells were sticking to components of the layer. Therefore, the results show that a cell culture system based on a simple Matrigel layer is not suitable for all kinds of experimental setups, for example, to investigate the adhesion of monocytes to EC.

We could also demonstrate significant changes in the secretion of the inflammatory markers depending on LD-RT with a specified IR dose. All three EC lines tested responded to various low doses of IR with a nonlinear dose-dependent secretion. These findings could be observed in both culture models. It is well known that high doses of IR result in proinflammatory reactions whereas low doses can cause both, anti-inflammatory and proinflammatory reactions. The effects of high- and low-dose IR on the immune system and the controversial results obtained with LD-RT not only in *in vitro* but also in *in vivo* experiments were summarized on the model of dendritic and T cell interaction [39].

Over the past decades, many other studies revealed the anti-inflammatory effect of LD-RT on various cells with different responses to the applied radiation doses, which were summarized by Rödel et al. [18].

The four markers examined are commonly known to be involved in inflammatory reactions caused by stimuli like IR [12] as well as TNF-*α* [45, 46]. It is already known that TNF-*α* has an effect on the vascular endothelium and on leukocyte interactions [47]. This proinflammatory cytokine may also be involved in the development of cardiovascular diseases, for example, atherosclerosis or congestive heart failure [48]. As suggested, the activation resulted in a distinctly higher release of inflammatory markers by all EC in both 2D and 3D cell culture systems compared to inactivated cells in our study. In a publication by Gerhardt et al. [48], an increase in proinflammatory markers like RANTES or MCP-1 by HUVECs after stimulation with a higher concentration of TNF-*α* (20 ng/mL) was observed. We could show this effect already with a 50% lower concentration (10 ng/mL). Another difference between the studies is the usage of a 3D-based cell culture model, in which we could demonstrate a much higher secretion of MCP-1 by EC compared to results obtained in the 2D model.

Additionally, to investigate a possible time- and dose-dependent change of cytokine expression on an mRNA level, qRT-PCR was performed with samples from mlEND.1 cells cultured under 2D as well as 3D conditions. It has already been revealed that various cells, cultured in both systems, respond to stimuli with an altered gene expression. IMR-90 cells (human fetal lung fibroblast cells) express genes like IL-8, CXCL1, CXCL2, or VEGF in higher levels under 3D culture conditions compared to samples from the 2D model whereas thrombospondin 1 (THBS1) was expressed at higher levels when cells were examined using the 2D model [49]. The metastatic melanoma cell line NA-8 also expressed various markers in different levels when cultured under 2D or 3D conditions. The gene expression of CXCL1, IL-8, and MIP-3 *α* was higher in 3D compared to samples of the 2D culture system also [22]. The expression pattern of MCP-1 in mlEND.1 cells was found to be noticeably different than protein secretion at the same time points and applied doses. Our findings suggest that MCP-1 underlies different posttranscriptional and posttranslational mechanisms, which could explain the diverging results obtained on a protein and mRNA level. In 2010, Shebl et al. analyzed various mRNA-protein correlations in PBMCs by testing 22 various cytokines. They illustrated a wide range of correlation between proteins secreted by the cells and the expression of the gene varying from marker to marker and also assumed the regulatory mechanisms after transcription and translation [50]. The transcription rates, splicing mechanisms, protein processing or degradation, or message turnover are just a few of many regulation mechanisms that can influence cytokine gene expression. Stimuli for inducing the expression are distinct and dependent on the cytokine. Possible mechanisms were summarized by Keene in 2007 where ribonucleoproteins (RNP) play a key role in mRNA processing, from transcription to protein synthesis [51].

A possible explanation for the different results detected in 2D and 3D might be the influence of the surrounding matrix, in which the cells are embedded. The ECM pore architecture and rigidity might affect the efficiency of growth factors and other molecules, like TNF-*α*, to diffuse through the environment. This influences the concentration gradient as well as the protein matrix binding. In the 2D model, cells are exposed to TNF-*α* almost completely, whereas cells in the 3D model are exposed to an attenuated concentration of the proinflammatory stimuli. This might affect the release and expression of inflammatory markers. Even the composition of the ECM will have an influence on not only the cell morphology and differentiation, but it may also affect the secretion of inflammatory markers. The manufacturer relinquished a vague content for the ECM used in this study which contains entactin, collagen IV, laminin, and heparin sulfate proteoglycan in different concentrations. It is well known that cytokines can interact or bind to components of the ECM, for example, proteoglycans [52]. Previously, the interaction of TNF-*α* with the ECM glycoproteins laminin (LN) and fibronectin (FN) was described, including how the interactions affected the activity of the proinflammatory protein [53]. Therefore, TNF-*α* was bound to both ECM components and possibly strengthened the binding of, for example, lymphocytes, to the ECM and promoting cell activation.

In the present paper, we focused on the radiation-induced effect of very low radiation doses because of clinical efforts to further dose reduction in LD-RT to minimize stochastic effects and possible carcinogenic late risks of LD-RT. For decades, LD-RT has been successfully applied using fraction doses of 0.5–1.0 Gy (total doses of 3–6 Gy). However, an optimal RT regimen is still not clear and under current discussion [54]. Additionally, due to discussion about a possible carcinogenic late risk, the application of LD-RT is still a subject of controversial debate and less accepted in many countries [55, 56]. For these reasons, a reduction in radiation doses in LD-RT is an impact of many efforts both clinically and experimentally.

In the last years, there were performed several clinical trials to reduce radiation doses in the treatment of inflammatory and painful joint diseases [1, 54, 57, 58]. According to these results, a new standard for the radiotherapeutical treatment of, for example, painful heel spur, was established with lowered fraction doses.

Recent laboratory studies have shown that single doses <0.1 Gy might be also effective for the treatment of benign diseases. It could be demonstrated that in vivo LD-RT has an impact on the functional as well as quantitative parameters of murine splenocytes [59]. A moderate decrease in the apoptosis of murine dendritic cells after whole body irradiation with low doses of 0.01–0.1 Gy was found. These observations were likewise associated with alterations of the cytokine milieu, including partial downregulation of IL-4 and IFN-*γ*. Liu et al. showed a stimulated expression of CD80 and CD86 on murine APCs after whole body irradiation with 0.075 Gy and increased IL-12 secretion 4 hours after IR [60]. Additionally, they were able to demonstrate that the expression of CD28 on T cells was upregulated and that of CTLA-4 was downregulated in early time points, also after very low radiation doses (0.075 Gy). In our study, we also could show that immune modulatory effects can be observed after irradiation with very low doses too. These findings may contribute to support of clinical efforts for further dose reduction to minimize stochastic effects and possible carcinogenic late risks of LD-RT.

## 5. Conclusions

Inflammation is a complex mechanism, and two important functions are the regulation of leukocyte migration and activation by cytokines. EC play a key role in these processes. After activation with appropriate stimuli, they can produce different adhesion molecules and inflammatory mediators like cytokines and chemokines. Little is known about the immune modulatory effects after LD-RT and the effects of these on EC function.

The results obtained in this study indicate an immune modifying ability of LD-RT regarding the response of EC not only in a conventional 2D culture system but also in an ECM-based 3D model. It has been clearly observed that the change of inflammatory cytokine release and monocyte binding to ECs was reliant on the origin of the EC, the radiation dose applied, and the time point after irradiation as well as TNF-*α* stimulation. For the proinflammatory cytokines KC, MCP-1, and RANTES, a dose-dependent concentration could be observed. LD-RT and/or TNF-*α* activation resulted in both reduction and increase of the cytokine levels. The monocyte adhesion was significantly enhanced after IR as well as activation. In the present study, the altered release of inflammatory cytokines was detectable in both culture models, 2D and 3D, but with clear differences.

With the results of the present study on the basis of EC, a better insight into the modulation of inflammatory reactions after LD-RT could be given. It was shown that 3D cultivation conditions are not only suitable but also advantageous for the investigation of modulation of inflammatory reactions after LD-RT and can be used for further studies.

## Data Availability

Data supporting this study are provided in the results section or as supplementary information accompanying this paper. Further datasets used and/or analyzed during the current study are available from the authors at the University Medical Center Rostock on request.

## Abbreviations

2D: Two-dimensional
3D: Three-dimensional
BBB: Blood-brain barrier
bFGF: Basic fibroblast growth factor
cDNA: Complementary deoxyribonucleic acid
CFSE: Carboxyfluorescein succinimidyl ester
DMEM: Dulbecco's modified Eagle's medium
EC: Endothelial cells
ECM: Extracellular matrix
FCS: Fetal calf serum
FN: Fibronectin
GAPDH: Glyceraldehyde 3-phosphate dehydrogenase
G-CSF: Granulocyte-colony stimulating factor
GPx: Glutathione peroxidase
Gy: Gray
HCEC: Human corneal epithelial cells
hCMVEC: Human cerebral microvascular endothelial cells
HLEC: Human lymphatic endothelial cells
HUVEC: Human umbilical vein endothelial cells
IL: Interleukin
Ig: Immunoglobulin
IL: Interleukin
IR: Ionizing radiation
KC: Keratinocyte-derived chemokine
LDL: Low-density lipoprotein
LD-RT: Low-dose radiotherapy
LN: Laminin
LPS: Lipopolysaccharide
MCP-1: Monocyte chemoattractant protein-1
mRNA: Messenger ribonucleic acid
NF*κ*B: Nuclear factor kappa B
RNA: Ribonucleic acid
qRT-PCR: Quantitative real-time polymerase chain reaction
PA: Plasminogen activator
PBMC: Peripheral blood mononuclear cells
PBS: Phosphate-buffered saline
PMN: Polymorphonuclear leukocytes
RANTES: Regulated on activation, normal T cell expressed and secreted
RNA: Ribonucleic acid
RNP: Ribonucleoprotein
SOD: Superoxide dismutase 1
THBS1: Thrombospondin 1
TH2: T-helper cell subtype 2
TNF-*α*: Tumor necrosis factor alpha
WST-1: Water-soluble tetrazolium
XIAP: X-linked inhibitor of apoptosis protein.

## Supplementary Materials

Supplementary Materials Supplement Figure 1: influence of low-dose irradiation on metabolic activity of murine endothelial cells, (A) without TNF-*α* induction and (B) with TNF-*α* induction. The cells were irradiated with different doses; a WST-1 assay was performed at three time points after irradiation. The extinctions were normalized to samples of 0 Gy/4 hours. Error bars present the standard deviation (±SD) from three independent experiments; wells were assayed in triplicates in each of the different experiments. Supplement Figure 2: released levels of the keratinocyte-derived chemokine (KC) in supernatant of (A) mlEND.1 and (B) bEND.3 endothelial cells. The cytokine concentration was determined by multiplex assay at two time points after irradiation with low doses of X-rays. Changes in cytokine concentrations are presented as mean (pg/mL) ± standard deviation (SD) from three independent experiments; asterisks illustrate significance: ^∗^ *p* < 0.05 and ^∗∗^ *p* < 0.01. Supplement Figure 3: released levels of monocyte chemoattractant protein-1 (MCP-1) in supernatant of (A) H5V and (B) mlEND.1 endothelial cells. The cytokine concentration was determined by multiplex assay at two time points after irradiation with low doses of X-rays. Changes in cytokine concentrations are presented as mean (pg/mL) ± standard deviation (SD) from three independent experiments; asterisks illustrate significance: ^∗^ *p* < 0.05, ^∗∗^ *p* < 0.01, and ^∗∗∗^ *p* < 0.001. Supplement Figure 4: released levels of RANTES in supernatant of (A) H5V and (B) bEND.3 endothelial cells. The cytokine concentration was determined by multiplex assay at two time points after irradiation with low doses of X-rays. Changes in cytokine concentrations are presented as mean (pg/ml) ± standard deviation (SD) from three independent experiments; asterisks illustrate significance: ^∗^ *p* < 0.05 and ^∗∗^ *p* < 0.01. Supplement Figure 5: released levels of G-CSF in supernatant of (A) 2D- and (B) 3D-cultured endothelial cells. The cytokine concentration was determined by multiplex assay at two time points after irradiation with low doses of X-rays. Changes in cytokine concentrations are presented as mean (pg/mL) ± standard deviation (SD) from three independent experiments; asterisks illustrate significance: ^∗^ *p* < 0.05.
