# Supplementary material for: Modulation of Inflammatory Reactions by Low-Dose Ionizing Radiation: Cytokine Release of Murine Endothelial Cells Is Dependent on Culture Conditions
Source: J Immunol Res. 2018 Jun 3;2018:2856518. doi: 10.1155/2018/2856518 (PMC6008836; doi:10.1155/2018/2856518)

Supplement Figure 1

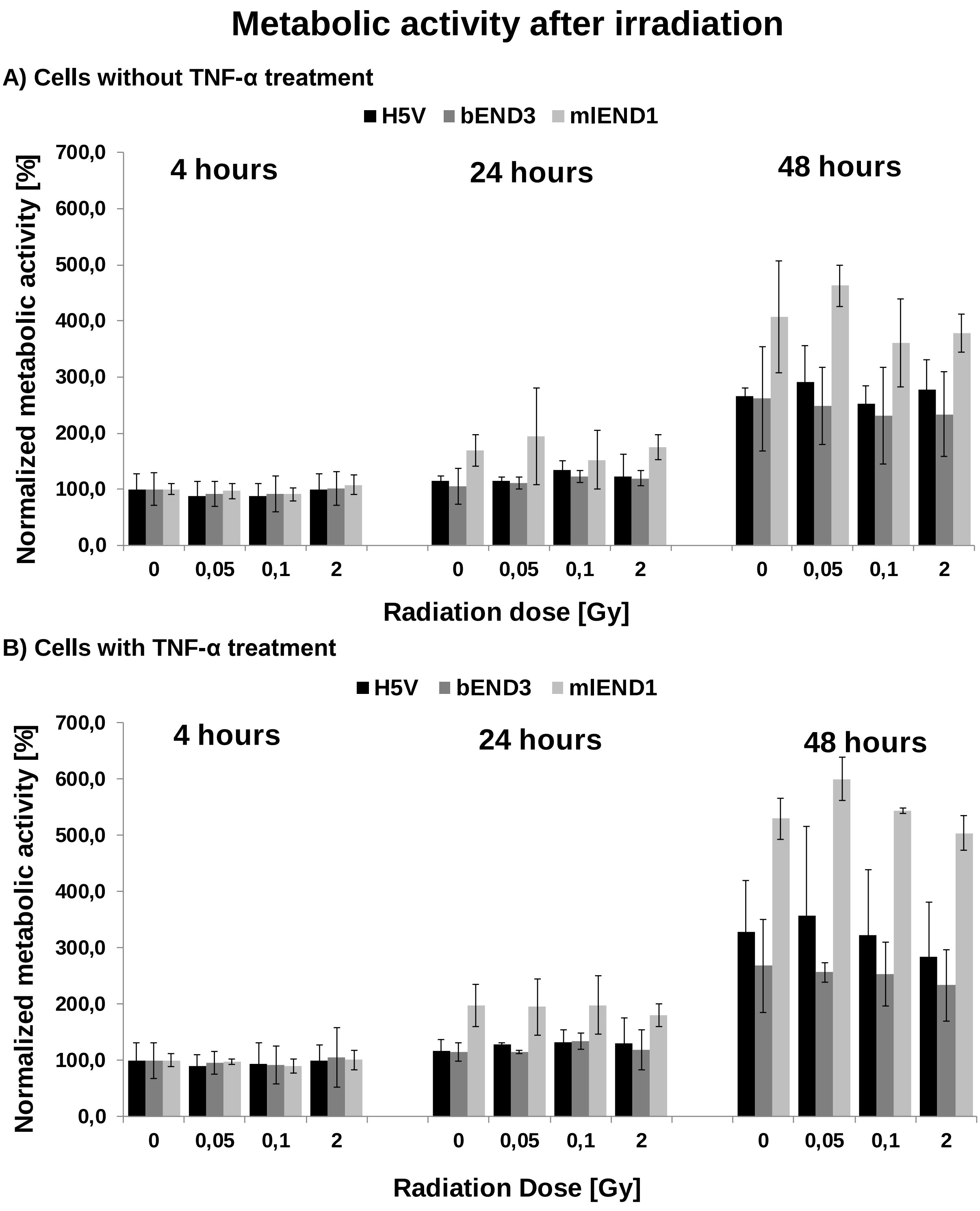

Supplement Figure 2

Release of KC after irradiation

A) mIEND.1 endothelial cells

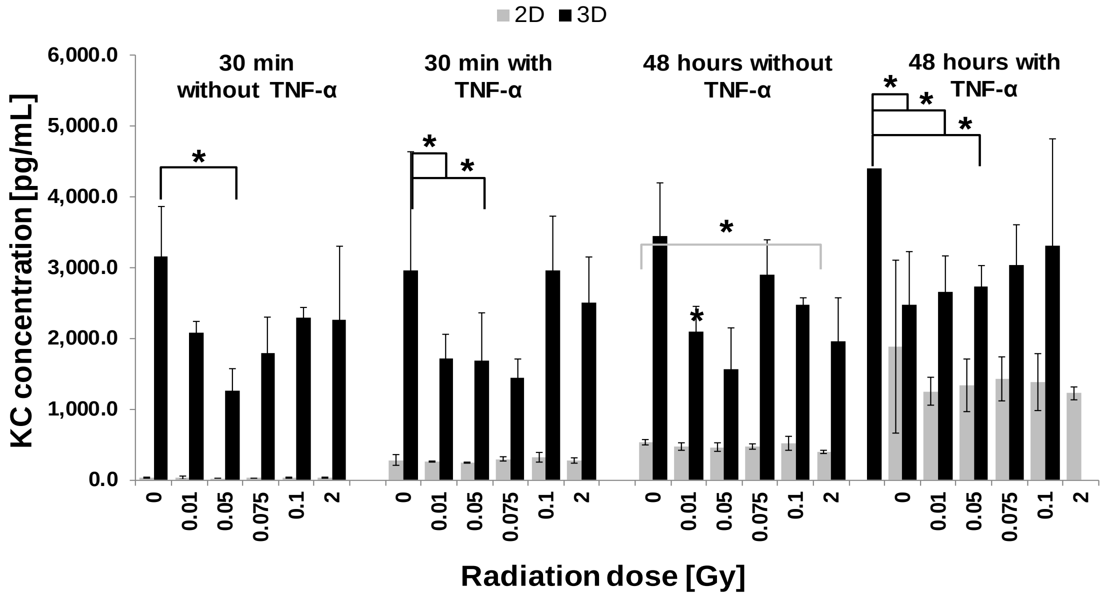

B) bEND.3 endothelial cells

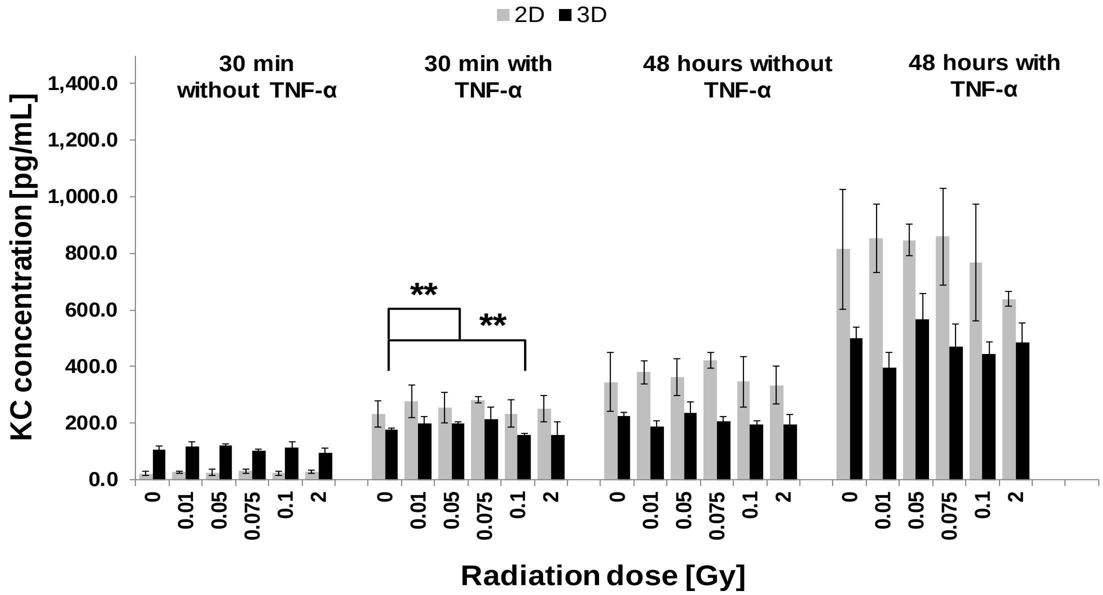

Supplement Figure 3

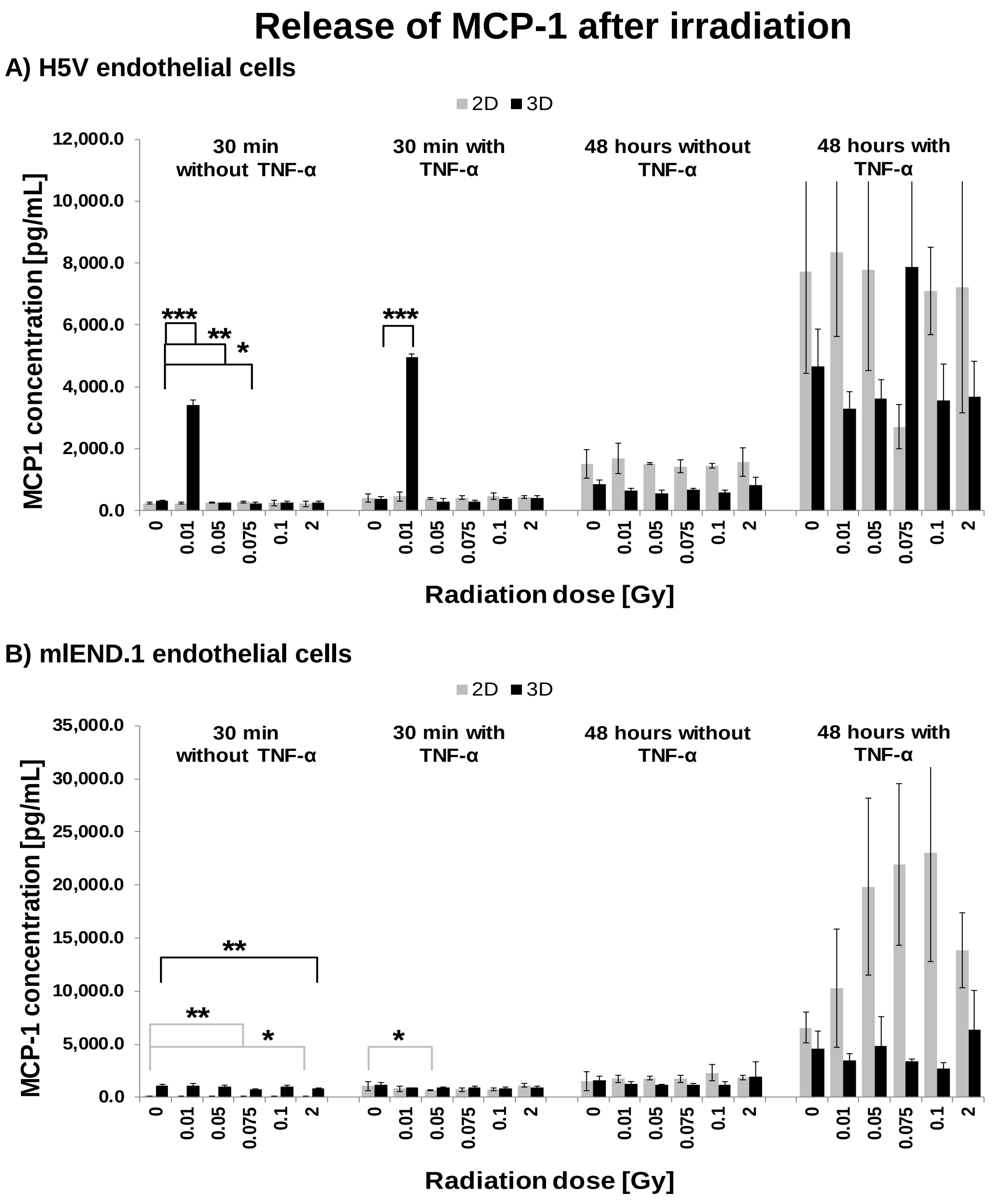

Supplement Figure 4

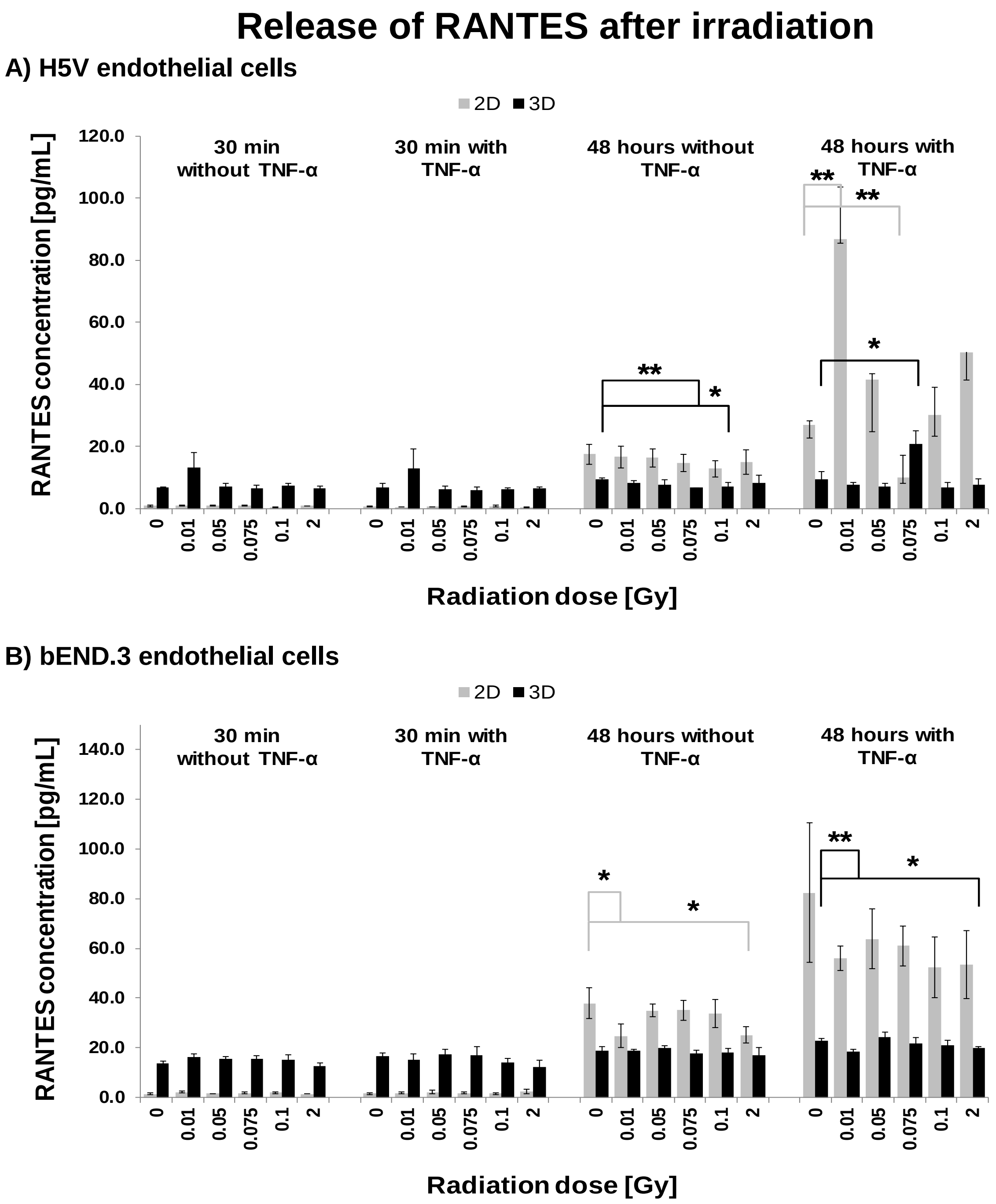

Supplement Figure 5

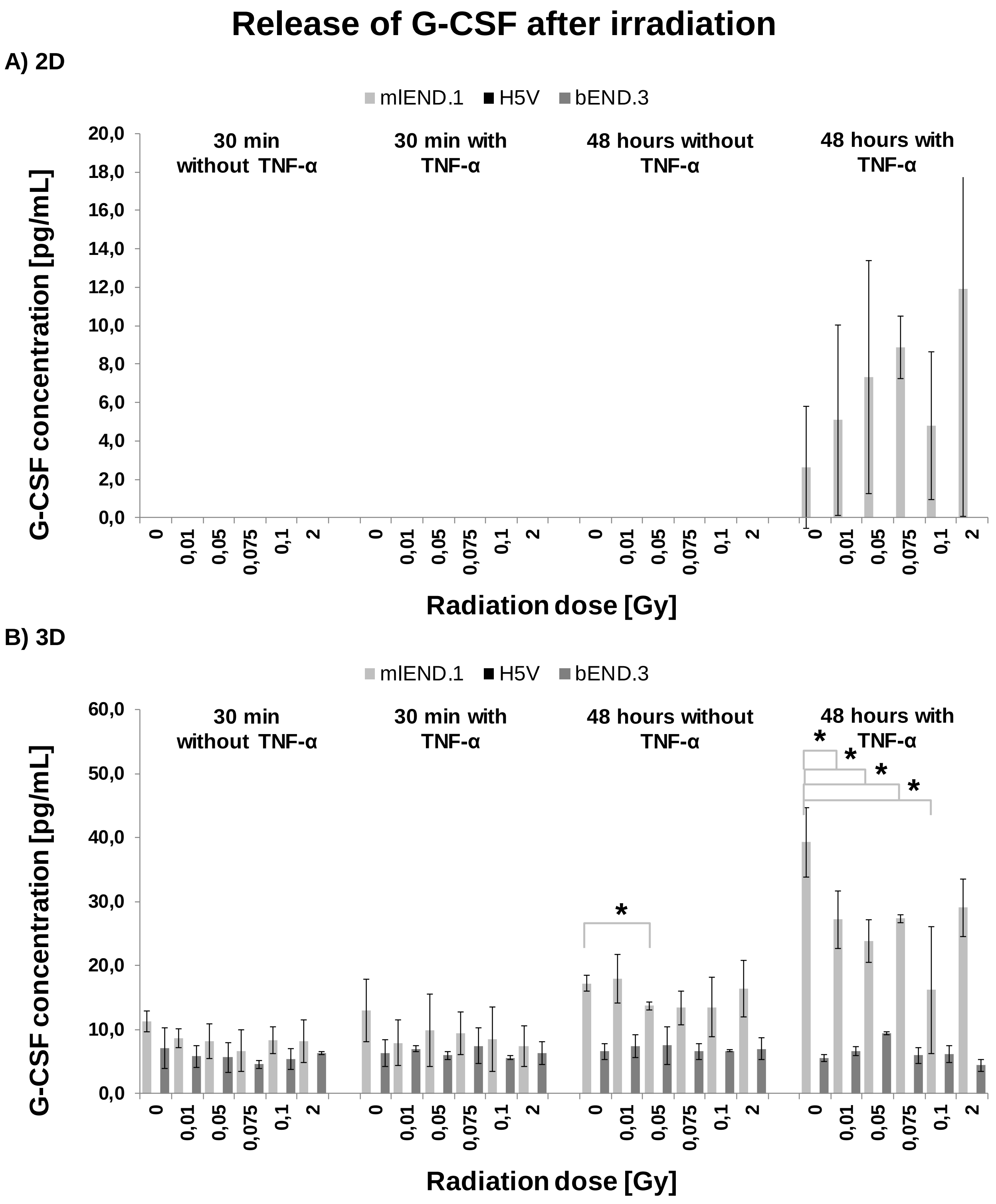

Supplement: Supplementary Materials — Supplement Figure 1: influence of low-dose irradiation on metabolic activity of murine endothelial cells, (A) without TNF-α induction and (B) with TNF-α induction. The cells were irradiated with different doses; a WST-1 assay was performed at three time points after irradiation. The extinctions were normalized to samples of 0 Gy/4 hours. Error bars present the standard deviation (±SD) from three independent experiments; wells were assayed in triplicates in each of the different experiments. Supplement Figure 2: released levels of the keratinocyte-derived chemokine (KC) in supernatant of (A) mlEND.1 and (B) bEND.3 endothelial cells. The cytokine concentration was determined by multiplex assay at two time points after irradiation with low doses of X-rays. Changes in cytokine concentrations are presented as mean (pg/mL) ± standard deviation (SD) from three independent experiments; asterisks illustrate significance: ∗ p < 0.05 and ∗∗ p < 0.01. Supplement Figure 3: released levels of monocyte chemoattractant protein-1 (MCP-1) in supernatant of (A) H5V and (B) mlEND.1 endothelial cells. The cytokine concentration was determined by multiplex assay at two time points after irradiation with low doses of X-rays. Changes in cytokine concentrations are presented as mean (pg/mL) ± standard deviation (SD) from three independent experiments; asterisks illustrate significance: ∗ p < 0.05, ∗∗ p < 0.01, and ∗∗∗ p < 0.001. Supplement Figure 4: released levels of RANTES in supernatant of (A) H5V and (B) bEND.3 endothelial cells. The cytokine concentration was determined by multiplex assay at two time points after irradiation with low doses of X-rays. Changes in cytokine concentrations are presented as mean (pg/ml) ± standard deviation (SD) from three independent experiments; asterisks illustrate significance: ∗ p < 0.05 and ∗∗ p < 0.01. Supplement Figure 5: released levels of G-CSF in supernatant of (A) 2D- and (B) 3D-cultured endothelial cells. The cytokine concentration was determine [file 2856518.f1.pdf]
